# Role of Parenting Style in Children’s Behavioral Problems through the Transition from Preschool to Elementary School According to Gender in Japan

**DOI:** 10.3390/ijerph16010021

**Published:** 2018-12-21

**Authors:** Rikuya Hosokawa, Toshiki Katsura

**Affiliations:** 1 School of Nursing, Nagoya City University, Nagoya 467-8601, Japan; 2 Graduate School of Medicine, Kyoto University, Kyoto 606-8501, Japan; tkatsura@hs.med.kyoto-u.ac.jp

**Keywords:** authoritarian parenting, permissive parenting, internalizing problem, externalizing problem, gender, preschool children

## Abstract

While ineffective discipline can be attributed to authoritarian and permissive parenting styles, little research has examined the role of gender in the association between parenting style and early childhood behavioral problems. Thus, this study aimed to clarify the effects of authoritarian and permissive parenting on children’s externalizing and internalizing behaviors during the preschool-to-elementary-school transition according to gender in Japan. A sample of 1668 Japanese children (853 boys and 815 girls) were followed longitudinally over one-year intervals, and assessed based on parenting styles (the Parenting Scale), children’s behavioral problems (the Strengths and Difficulties Questionnaire), and family characteristics. Multivariate analyses revealed that, when analyzed by gender, authoritarian discipline influenced externalizing problems in boys (β = 0.048, *p* = 0.047) and girls (β = 0.067, *p* = 0.023), while permissive discipline influenced externalizing problems in boys only (β = 0.049, *p* = 0.038). The results document the relationship between family processes and the development of disruptive behavior disorders in children. Support for parents employing such child-rearing styles in early childhood may be effective in reducing school maladjustment.

## 1. Introduction

Behavioral problems including externalizing problems (e.g., hyperactivity, rule-breaking behaviors, and aggression) and internalizing problems (e.g., anxiety, withdrawal, and depression) are among the most common mental health issues in early childhood [1,2,3]. Moreover, behavioral problems in early childhood are a significant predictor of negative mental health outcomes in later life [4,5,6]. For example, they are highly predictive of a range of difficulties during childhood, such as conduct disorder, poor scholastic achievement, and peer rejection [7,8,9], as well as negative outcomes in adolescence and adulthood, such as mental health problems, poor employment prospects, and antisocial behavior [10,11,12]. During early childhood, several developmental trajectories emerge [13,14]. Behavioral problems in early childhood may also hinder the development of a range of social, emotional, and cognitive competences in children [15,16], and early childhood is a critical period for the development of behavioral problems related to social maladaptation. Finally, untreated behavioral problems during early childhood are considered disadvantageous [17,18,19]. Thus, to prevent the development of behavioral problems, it is necessary to understand the early childhood development of these problems as well as their etiology and developmental course.

Early childhood child-rearing environments can significantly affect the incidence of children’s behavioral problems. The family is a social arrangement that exerts significant influence on children’s development and parenting styles, and in particular, influences the social environments of children within the family. Moreover, parenting is an ecological variable that significantly influences a child’s personality development [20,21,22]. Some previous studies have suggested that maternal and paternal parenting styles play a central role in understanding the development of externalizing and internalizing behaviors [23,24], since parenting style exacerbates or reduces children’s behavioral problems [25,26,27]. The effects on development of parenting behaviors in childhood tend to be stable over time [28,29]. Depending on the parenting style they adopt, parents elect to use different discipline strategies to manage their children’s behavior and such strategies are considered a fundamental aspect of parenting [30].

The most frequent categorization of ineffective discipline includes the two styles of parenting identified by Baumrind—the authoritarian and permissive styles—each of which is theorized to differentially influence offspring [31,32,33]. According to Baumrind’s typology, parenting styles vary on a two-dimensional framework of parental demandingness (e.g., control, supervision, maturity demands), which refers to the role parents play in promoting respect for rules and social conventions, and responsiveness (e.g., warmth, acceptance, involvement), which is related to the level of parental involvement between children and parents. Ineffective discipline can be attributed to two parental authority prototypes or styles, authoritarian and permissive, which are characterized as being either high or low on the two parenting dimensions (i.e., demandingness and responsiveness).

The authoritarian style has high demandingness and low responsiveness and is characterized by firmly enforced rules and edicts decided by parents as well as strong control over children’s autonomy [34,35,36,37]. Parents with an authoritarian style attempt to control the behavior of their children in accordance with a set standard of conduct, usually an absolute standard [33]. Authoritarian parents tend to use demands to discipline their children and only allow them minimal autonomy. They also demonstrate low affection and emotional warmth in their parent–child relationships [36,37]. This type of parenting is likely to be negatively associated with children’s psychosocial development; that is, children of authoritarian parents are likely to have low self-esteem, be less content and less secure, and have negative attitudes toward the world; conversely, they are likely to do well in terms of obedience to standards [37,38].

Permissive parenting, on the other hand, has low demands and high responsiveness. It is characterized by a lack of monitoring, control, and discipline, yet it is warm and nurturing [39,40]. Permissive parents attempt to behave in a nonpunitive and accepting manner toward their children’s desires and actions and allow their children to regulate their own activities as much as possible [33]. Permissive parenting occurs when parents fail to set limits and do not expect developmentally appropriate behavior of their children. As a consequence, this type of parenting is likely to be negatively associated with children’s psychosocial development and children of permissive parents exhibit characteristics such as narcissistic tendencies, social irresponsibility, and self-centered motivation [41,42]. Consequently, both authoritarian and permissive disciplinary styles are potentially harmful for children’s psychosocial development. Furthermore, such parental styles have been linked consistently with a variety of negative developmental outcomes including behavioral problems over time [43,44,45,46,47,48,49].

Although several studies focusing on parenting styles and behavioral problems and involving mixed samples (i.e., including both boys and girls) have linked ineffective discipline to disruptive behavioral problems in children, these studies did not differentiate between boys and girls. That is, the research has not sufficiently examined how parenting styles influence child outcomes within the context of child gender dyads. Children’s gender has been found to be important in the emergence of child behavior problems and how children respond to parenting styles, and several studies have suggested the role of gender differences in internalizing versus externalizing problems [50,51,52,53,54,55,56]. Early childhood is a critical period for child development related to social adaptation. Because gender can play a role in discipline-associated outcomes in early childhood, it is important to clarify the effects of authoritarian and permissive parenting on behavioral problems according to gender in this period. Furthermore, there is consistent evidence with mixed samples in North American and European families that authoritarian and permissive parenting in early childhood are associated with poor child outcomes. Although it has been debated whether these relationships can be generalized to families in East Asian cultures, several studies using East Asian subjects have produced similar results to those using Western subjects. For instance, concerning school performance, one study focusing on Chinese children (mean age 7 years) demonstrated a negative effect of authoritarian parenting [57]. Another study on Chinese children (mean age 9 years) demonstrated that authoritarian parenting led to low self-regulation and learning problems in children [58]. However, while many studies have shown the cultural differences between East Asian and Western parenting styles, there has been limited research on the effect of authoritarian and permissive parenting on behavioral problems in early childhood in Japan. Japanese and Chinese parents are more likely to endorse an authoritarian parenting style, such as strictness and parental control, than parents from Western cultures [59,60]. Moreover, children in East Asian countries are more likely to be required to be obedient and polite than those in Western countries [61,62]. Thus, cultural differences can play a role in discipline-associated child outcomes in early childhood and it is important to clarify the effects of authoritarian and permissive parenting on behavioral problems according to gender in early childhood in Japan.

In summary, authoritarian and permissive parenting directly impact children’s behavioral problems. Although many studies have focused on this relationship, to date, not enough research has examined the association between parenting style and children’s behavioral problems in early childhood, with a focus on the role of child gender. In Japan specifically, limited research has examined the effect of authoritarian and permissive parenting style in early childhood on behavioral problems. Therefore, the present study attempts to clarify the effects of authoritarian and permissive parenting respectively in early childhood on the externalizing and internalizing problem behaviors of children in elementary school, focusing on gender, in Japanese children. The influence of different negative parenting styles in preschool on the presence of externalizing and internalizing problem behaviors in elementary school could differ between boys and girls. We hypothesized that in a mixed sample using Japanese subjects, authoritarian and permissive parenting in early childhood would be associated with poor child outcomes, producing similar results to studies conducted in other countries. We also hypothesized that authoritarian parenting would be more strongly associated with behavioral problems in girls than in boys and that permissive parenting would be more strongly associated with behavioral problems in boys than in girls.

## 2. Materials and Methods

### 2.1. Participants

The children targeted by this study, all of whom were five years old, were recruited from 52 kindergartens and 78 nursery schools. Recruitment was conducted in 2014 in Nagoya City, which is a major urban area in Aichi Prefecture in Japan. The targeted children and their parents participated in the longitudinal project designed to examine the influence of family factors on children’s social developmental outcomes. The first two cohorts (2014: T1; 2015: T2) of participants were included in this study. A total of 3314 of the 5024 target families completed the baseline assessment (T1), and of these, 1787 completed both the baseline (T1) and follow-up assessments (T2), indicating a retention rate of 53.9%.

To recruit participants, self-report questionnaires were distributed to all parents of the targeted children (*n* = 5024) at T1 when the children were five years old and in preschool, and the response rate was 66.0% (*n* = 3314). At T2, when the children were six years old and in first grade, a second questionnaire was distributed to the families (*n* = 3314) and the response rate was 53.9% (*n* = 1787). Thus, the attrition rate from T1 to T2 was 46.1%, with 44.7% not responding at T2 and 1.4% (*n* = 46) having relocated.

There were a number of significant differences between those who participated in the baseline and one-year follow-up assessments (*n* = 1787) and those who did not respond to the T2 assessments (*n* = 1527), with the latter being more likely to have lower socioeconomic status (SES) (i.e., lower household income and parents with lower levels of education). A comparison of the demographic features between the non-returning and returning participants revealed that 16.2% of the non-returning participants’ annual household income was less than Ұ3,000,000, while this was true of only 10.0% of the returning participants; the household income of the non-returning participants was significantly lower than that of the returning participants, as measured by a chi-square test. In addition, 5.8% of the non-returning participants’ maternal educational background comprised compulsory education, while this was true of only 2.4% of the returning participants; furthermore, 7.6% of the non-returning participants’ paternal educational background comprised compulsory education, while this was true of only 4.7% of the returning participants. Both the maternal and paternal educational levels of non-returning participants were significantly lower than those of the returning participants, as measured by a chi-square test. Thus, the non-returning participants tended to have relatively lower SES than did returning participants, meaning that there was a lower response rate of individuals with low SES compared to those with high SES. Hence, those participants lost from the sample comprised families with higher rates of lower sociodemographic factors, meaning that these families were underrepresented in our sample and our results may be less generalizable to families with a lower socioeconomic background.

To accurately clarify the associations between parenting attitude and child developmental outcomes, we then chose to exclude children with developmental problems (*n* = 75) and children whose mothers did not complete question items regarding parenting attitude and child developmental outcomes (*n* = 44). Consequently, of the 1787 children for whom data were received at both T1 and T2, 1668 (93.3%) met the inclusion criteria.

### 2.2. Ethics Statement

The researchers obtained written, informed consent from all participants. Written, informed consent was obtained on behalf of the children from their parents. Ethical approval for this study was obtained from Kyoto University’s Ethics Committee in Kyoto, Japan (E2322). All procedures performed in studies involving human participants were in accordance with the standards of the ethics committee.

### 2.3. Measures

#### 2.3.1. Parenting Behavior: Parenting Scale (PS)

The Parenting Scale (PS) is a questionnaire that measures dysfunctional discipline styles in parents to manage children’s behavior [63]. The PS Japanese version was used in this study [64]. At the baseline assessment, parents completed the PS. Each of the 30 items is rated on a 7-point scale. Two subscales from the PS were used to assess dysfunctional discipline practices when faced with problem situations. Ratings of parental behavior are obtained with regard to ineffective discipline styles: laxness (i.e., permissive discipline) and over-reactivity (i.e., authoritarian discipline). The scale shows adequate internal consistency and also correlates closely with direct observations of mother–child interactions [63,64]. The Cronbach’s alphas for laxness and over-reactivity in this sample were 0.70 and 0.86, respectively; the scale demonstrated adequate internal consistency (see Table 1).

For the PS Japanese version, the items of over-reactivity were similar to those of the original version [63]. Regarding laxness, although the concept was similar, slight differences were observed in the items. Specifically, although the original version included “*If saying ‘No’ doesn’t work, I offer my child something nice so he/she will behave*” as one of the laxness items, the Japanese version did not include this item and instead included “*When my child pesters me, I can’t ignore the pestering*.” In Japanese, “laxness” is likely to mean that parents are at their children’s beck and call rather than that parents make a deal with children to temporarily handle the situation. Thus, adjustments to the PS were made to account for the difference between Japanese and Western permissive parenting styles.

#### 2.3.2. Child Behavioral Problems: The Strengths and Difficulties Questionnaire (SDQ)

The Strengths and Difficulties Questionnaire (SDQ) measures child behavioral problems [65]. The SDQ Japanese version was used in this study [66]. Parents completed the SDQ at both the baseline assessment (T1) and at the one-year follow-up assessment (T2). The SDQ is a 25-item measure of parents’ perceptions of prosocial and difficult behaviors in their children, designed to assess the frequency of positive and negative behaviors in children [65]. It comprises five subscales (i.e., emotional symptoms, conduct problems, hyperactivity, peer problems, and prosocial behavior), and the score for each subscale is computed by adding the scores for the five items. The difficult behavior score in the SDQ is calculated as the sum of the scores obtained on the emotional symptoms, conduct problems, hyperactivity, and peer problems subscales. In this study, the emotional symptoms and peer problems subscales of the SDQ were combined to form an internalizing problems scale, while the conduct problems and hyperactivity-inattention subscales were combined to form an externalizing problem scale, as suggested by Goodman et al. [67]. The scale has been validated as having adequate internal reliability [66]. In this sample, the Cronbach’s alphas for internalizing and externalizing problems were 0.65–0.71 and 0.74–0.77, respectively; the scale demonstrated adequate internal consistency (see Table 1).

#### 2.3.3. Demographic Covariates

Parents provided their demographic information, including child’s sex, family structure (single-parent or two-parent family), attending institution (kindergarten or nursery school), annual household income, maternal education level, and paternal education level. Regarding family income, parents reported their annual household income in Japanese yen (JPY). In this study, the median household income was between 5.00 and 5.99 million JPY, and the average number of people per household was 4.3. In Japan, the median income of a four-person household is approximately 6.00 million JPY, so the household income of participants in this study was relatively similar to the typical Japanese distribution. The threshold for four-person households in Japan, calculated based on the Organisation for Economic Co-operation and Development’s standard for the poverty line (i.e., half the median income of the total population), is an annual income of approximately 3.00 million JPY [68]. Therefore, with reference to those values, we created four categories of income: <3 million, 3–5 million, 6–8 million, and ≥9 million JPY. Regarding parental education, both parents were asked to report their education in years, as well as the highest level of education completed. The Japanese education system comprises elementary school (6 years), junior high school (3 years), and high school (3 years), and education is compulsory until the end of junior high school (9 years). Based on the data, we created four categories of education level: compulsory education (9 years), upper secondary school (12 years), up to four years of college/university (13–15 years), and four or more years of college/university (≥16 years). At the baseline assessment, parents provided complete information regarding these factors (see Table 2).

### 2.4. Data Analyses

First, the relationships between participant characteristics and children’s behavioral problems were analyzed via *t*-test or one-way analysis of variance (ANOVA) as shown in Table 3. Second, the effects of parenting practices during preschool (T1) on children’s outcomes in first grade (T2) were assessed using multiple linear regression, with PS scores of laxness (i.e., permissive discipline) and over-reactivity (i.e., authoritarian discipline) as predictors and SDQ scores of internalizing or externalizing problems as the outcome (see Table 4, Table 5 and Table 6). Table 4 shows the entire sample of both boys and girls (*n* = 1668), Table 5 shows only boys (*n* = 853), and Table 6 shows only girls (*n* = 815). Multivariate regression analyses were performed as follows: in Models 1–2, each predictor was entered individually to assess its univariate association with each outcome (i.e., laxness (Model 1), over-reactivity (Model 2)). In Model 3, both predictors (i.e., laxness and over-reactivity) were entered simultaneously. As several participant characteristics at T1 were significantly associated with behavioral problems at T1 in the analyses (see Table 3), we included the factors as covariates in each analysis (see Table 4, Table 5 and Table 6). Multicollinearity among the predictors was assessed, and no problems were found. IBM SPSS Statistics 23.0 (IBM Corporation, New York, NY, USA) was used for all statistical analyses with a significance level of 5%.

## 3. Results

### 3.1. Study Population

Participants’ demographic characteristics at preschool (i.e., at baseline) are shown in Table 2, and the relationships between participants’ demographic characteristics and emotional/behavioral problems at first grade are shown in Table 3. The children’s average age was 6.09 years (SD = 0.30), with 51.1% boys (*n* = 853) and 48.9% girls (*n* = 815). Regarding family structure, in the mixed sample and girls sample, the internalizing problem scores of children from single-parent families were significantly higher than those of children from two-parent families; similarly, in the mixed sample and girls sample, externalizing problem scores of children from single-parent families were significantly higher than those of children from two-parent families. Regarding attending institution, in the mixed sample, nursery school children’s internalizing problem scores were significantly higher than kindergarten children’s internalizing problem scores; in the mixed sample, boys sample, and girls sample, nursery school children’s externalizing problem scores were significantly higher than kindergarten children’s externalizing problem scores. Regarding annual household income, in the mixed, boys, and girls samples, the internalizing problem scores of children from lower-income households were significantly higher than those of children from higher-income households; similarly, in the mixed, boys, and girls samples, the externalizing problem scores of children from lower-income households were significantly higher than those of children from higher-income households. Regarding maternal education level, in the mixed, boys, and girls samples, the internalizing problem scores of children from lower-income households were significantly higher than those of children from higher-income households; similarly, in the mixed, boys, and girls samples, the externalizing problem scores of children from lower-income households were significantly higher than those of children from higher-income households. Regarding paternal education level, in the mixed sample, the internalizing problem scores of children from lower-income households were significantly higher than those of children from higher-income households; in the mixed and boys samples, the externalizing problem scores of children from lower-income households were significantly higher than those of children from higher-income households.

### 3.2. Effect of Parenting Practices on Behavioral Problems

The associations between parenting practices and children’s outcomes were assessed using multiple linear regression, with PS scores of laxness (i.e., permissive discipline) and over-reactivity (i.e., authoritarian discipline) as predictors and SDQ scores of internalizing or externalizing problems as the outcome (see Table 4, Table 5 and Table 6). First, regarding analysis targeting boys and girls (*n* = 1668), the results of the multivariate analysis between parenting practices and behavioral problems are shown in Table 4. Regarding internalizing problems as the outcome, in the models that included individual predictors (i.e., Models 1 and 2), each predictor (i.e., laxness and over-reactivity) was not significantly associated with internalizing problems. Further, in Model 3 (which contained both predictors), no predictor was significantly associated with internalizing problems. On the other hand, regarding externalizing problems as the outcome, in Models 1 and 2, each predictor was significantly associated with externalizing problems. Further, in Model 3, both laxness (β = 0.040, *p* = 0.034) and over-reactivity (β = 0.055, *p* = 0.005) were significantly associated with externalizing problems.

Second, regarding analysis targeting only boys (*n* = 853), the results of the multivariate analysis between parenting practices and behavioral problems are shown in Table 5. Regarding internalizing problems as the outcome, in Models 1 and 2, laxness and over-reactivity were not significantly associated with internalizing problems. Further, even in Model 3, these predictors were not significantly associated with internalizing problems. On the other hand, regarding externalizing problems as the outcome, in Models 1 and 2, each predictor was significantly associated with externalizing problems. Further, in Model 3, both laxness (β = 0.049, *p* = 0.038) and over-reactivity (β = 0.048, *p* = 0.047) were significantly associated with externalizing problems.

Finally, regarding analysis targeting only girls (*n* = 815), the results of the multivariate analysis between parenting practices and behavioral problems are shown in Table 6. Regarding internalizing problems as the outcome, in Models 1 and 2, laxness and over-reactivity were not significantly associated with internalizing problems. Further, even in Model 3, both these predictors were not significantly associated with internalizing problems. On the other hand, regarding externalizing problems as the outcome, in Models 1 and 2, over-reactivity but not laxness was significantly associated with externalizing problems. Further, in Model 3, laxness was not significantly associated with externalizing problems, whereas over-reactivity was significantly associated with externalizing problems (β = 0.067, *p* = 0.023).

## 4. Discussion

Based on our longitudinal observation, the findings of the present study highlighted the significant impact of parenting styles on children’s behavioral problems, focusing on the role of child gender. Our results demonstrate a clear relationship between discipline styles in preschool for five-year-old children and behavioral problems in first grade for six-year-old children. These findings are consistent with previous studies that suggest that authoritarian and permissive disciplinary styles are associated with disruptive behavioral problems [69,70,71,72,73]. Specifically, multivariate analyses in the mixed sample revealed that not only higher over-reactivity (i.e., high authoritarian discipline) but also higher laxness (i.e., high permissive discipline) predicted clinically significant externalizing problems. In addition, based on an analysis of gender, authoritarian discipline was found to be related to externalizing problems in both boys and girls, whereas permissive discipline was related to externalizing problems only in boys. It is important to note that in our study, the relationships between parenting and child behavioral problems were found after controlling for child characteristics as well as demographic characteristics such as family structure and family SES at baseline. As these analyses controlled for these variables, the relationships between parenting and child behavioral problems were independent of the effect of child and demographic characteristics. Many studies have also suggested that behavior development identifies major risk factors for childhood behavioral problems; for example, not only parenting techniques but also child characteristics and family adversities such as low SES and single parenting have been shown to increase the risk of developing childhood behavioral difficulties [74,75,76,77,78,79]. A finding of note in the present study was gender difference that permissive discipline was related to externalizing behavioral problems for boys but not girls. Several of the mechanisms involved in this relationship between disciplinary styles and the risk of behavioral problems are likely to differ among boys and girls in early childhood.

### 4.1. Effects of Authoritarian Parenting Style on Behavioral Problems

The current findings suggest that the authoritarian parenting style was detrimental for both boys and girls. This finding is consistent with previous studies that also suggest that the authoritarian parenting style is linked to externalizing problems [69,70,80,81,82,83]. However, although most previous studies have analyzed mixed samples, they did not differentiate between boys and girls. This study, however, found that authoritarian discipline predicted clinically significant externalizing problems for both boys and girls. There was no gender difference in this relationship. Authoritarian discipline encompasses a restrictive style of interaction with children, which does not take the children’s views and wishes into account. Authoritarian parents can influence the behaviors of their children in society, who tend to be unsuccessful because prohibition and power assertion are likely to be related to anxiety, fear, and frustration in children; moreover, such parents are likely to be subjected to their children’s misbehaviors and other psychosocial behaviors. Indeed, children of parents with high-control parenting styles such as power-assertive, prohibitive, and punitive strategies have been shown to be less content, less secure, and more likely to become hostile or regressive and have greater difficulties dealing with somatic distress or psychological issues when under high stress than other children [84,85]. Moreover, coercive parenting styles are likely to prevent children from learning to self-regulate their own behaviors [86,87]. In addition, lack of parental warmth is also a risk factor for aggressive and disruptive behavioral problems in children [88] and could contribute to the development of deviant behaviors and adjustment problems. Therefore, in this study, children who received authoritarian parenting including high control and low warmth appear to be at an increased risk of externalizing behavioral problems.

Authoritarian parenting style tends to be more common in Japan than in Western countries [64], perhaps due to parents’ expectations or environmental conditions. In Japan, children are required to be more obedient and polite than in Western countries [61]. For instance, when children make noise, parents may overreact from worry about bothering others. Therefore, parents in Japan may be more severe than Western parents in punishing bad behaviors and thus use a more control-oriented parenting style. Although we hypothesized that authoritarian parenting was more associated with behavioral problems in girls than in boys, we found that it was associated with behavioral problems in both boys and girls, perhaps influenced by the Japanese cultural background.

### 4.2. Effects of Permissive Parenting Style on Behavioral Problems

Permissive parenting was found to be related to disruptive behavior in boys but not in girls. Permissive parents dislike having control and authority over their children and therefore do not guide them to regulate their behaviors and allow them to make decisions alone. Because they are not comfortable imposing restrictions on their children, they tend to avoid and tolerate their children’s misbehavior. This type of parenting style can allow the children to control their parents through coercion and thus indulges their low self-control and aggressive behaviors [41,42,89]. Consequently, it is assumed that the permissive parenting style will be positively associated with children’s externalizing behavioral problems.

In this study, there was significant gender difference; permissive discipline was related to externalizing behavioral problems in boys but not in girls. Some sources suggest that non-permissive (i.e., firm) disciplinary strategies may be particularly important primarily for boys. For instance, the early parenting model for the development of disruptive behavior focuses primarily on boys and emphasizes the importance of non-permissive discipline to deal with disruptive behaviors [90,91]. The model focusing on gender in early intervention programs suggests that permissive discipline may be particularly detrimental for boys, perhaps because boys have less well-developed self-regulation processes than similarly aged girls [92,93]. This may also be attributed to the gender differences among children in accepting parenting style. How children rate fathers’ or mothers’ parenting styles may depend on the child’s gender. A previous study suggested that boys perceived permissive parenting more than girls [94]. In addition, boys were likely to rate permissive styles less positively than girls [95]. These tendencies can more strongly influence the effect of permissive parenting on outcomes for boys than for girls. This study also found that respondents on parenting style were mothers. Boys, rather than girls, were more likely to rate mothers as permissive [94]. Thus, in this study, boys were more influenced by the permissive discipline of their mothers and in turn may be more exposed to the risk of behavioral problems from permissive parenting than girls. Therefore, in this study, there was likely to be gender difference; permissive discipline was related to externalizing behavioral problems in boys but not in girls.

On the other hand, as mentioned in the Materials and Methods section, although in the present study, the concept of laxness (i.e., permissive discipline) was similar in the PS Japanese version to the original version, slight differences were observed in the items. In Japanese, “laxness” is used to mean that parents are at their children’s disposal rather than that parents negotiate with children to temporarily manage a given situation. Thus, the Japanese permissive parenting style may differ from Western countries’ version thereof, meaning that this trend (i.e., permissive discipline’s relation to externalizing behavioral problems in boys but not in girls) may differ in Western countries.

### 4.3. Limitations

There are several limitations to this study, which need to be addressed in future research. First, this study was designed as an observational study. It is reasonable to assume that children’s problem behaviors interact with parenting styles in predicting developmental outcomes. Many studies have reported that there are bidirectional relationships between children’s conduct problems and negative parenting behaviors such as the use of harsh and coercive disciplinary strategies [70,96,97]. Negative parenting practices have often interacted with child oppositional and aggressive behavior, such that parents of children with behavioral problems were found to practice more negative parenting styles than parents of children without such problems [96,97]. Although this study included child behavioral problems as a baseline survey (T1) and a follow-up survey (T2), parenting style was only included in the baseline survey and therefore the transactional influence could not be estimated. Furthermore, although this study included child and family characteristics as moderator variables in the baseline survey to control for interaction effects, we did not include other multifaceted moderator variables. Therefore, further explorations should include parenting style in both the baseline and follow-up surveys and more multifaceted moderator variables or should conduct an intervention study.

Second, the present study did not include genetic factors. Child temperament has been conceptualized as a pathway through which children’s genotypes affect parenting style because child temperament is genetically influenced [98]. For instance, children’s negative emotionality was moderately associated with parental negativity and accounted for some part of the total child-based genetic contributions to parental negativity [99]. Further study should use a genetically informed design to include genetic factors; alternatively, twin studies could be conducted.

Third, in this study, to assess child behavioral problems, the SDQ was completed by parents only, which likely introduced reporting bias. Teachers’ reports might be needed to evaluate this more accurately and further explorations should combine teacher as well as caregiver SDQ ratings. Furthermore, in this study, only mothers were asked about their parenting styles and the parenting styles of mothers and fathers are likely to differ. Apart from the fact that the quantity of time fathers and mothers spend with their children is different, there are indications that parental involvement is also qualitatively different; for instance, mothers were more likely to use an authoritative style, whereas fathers were more likely to use an authoritarian style [100,101]. In addition, children pick up parenting style differently from fathers and mothers; for instance, boys were more likely than girls to rate mothers as permissive and fathers as authoritarian, while girls tended to rate their mothers as more authoritative and fathers as more permissive [94]. Thus, the parenting styles of fathers and mothers are likely to differ, while boys and girls are likely to have different views on their mothers’ and fathers’ parenting styles. In future research, it is necessary to grasp the child-rearing styles of both fathers and mothers.

Finally, these findings may not be generalizable to all families due to a risk of attrition bias. The retention rate from T1 to T2 was 53.9%, and the returning participants in T2 tended to have relatively higher SES than the non-returning participants, meaning that families with lower SES were underrepresented in our sample, and our results may be less generalizable to families with a lower socioeconomic background. This is a relatively universal problem in parenting research in general—parenting is sufficiently stressful that it is difficult to get the population of parents to participate. Future research would benefit from a study design using samples with higher retention rates (in particular, participants with lower SES).

## 5. Conclusions

This longitudinal study of a large sample of Japanese children found evidence that, during the important transition period between preschool and elementary school (age five to six), parenting style is associated with children’s behavioral problems at age six. Authoritarian and permissive parenting styles are likely to continue to negatively influence children’s development beyond childhood. In addition, authoritarian discipline was related to disruptive behaviors in both boys and girls, whereas permissive discipline was related to disruptive behaviors in boys but not in girls. The permissive parenting style will be associated positively with externalizing behavior in boys rather than girls because of gender deference. Few studies have examined the way parental authority influences the later development of behavioral problems in children, specifically with a focus on gender. These findings add to the substantial literature documenting the relationship between family processes and the development of disruptive behavior disorders in early childhood, specifically with a focus on gender. Therefore, support for parents that focuses on the negative effects of such child-rearing styles in early childhood according to child gender may be effective in preventing school maladjustment.

## Figures and Tables

**Table 1:** Descriptive statistics for the study variables (*n* = 1668).

Description	Range	Mean	SD	α
Parenting practices: Parenting Scale (PS)				
Laxness	1–7	2.53	0.78	0.70
Over-reactivity	1–7	3.62	1.10	0.86
Child adjustment: Strengths and Difficulties Questionnaire (SDQ)				
Internalizing problems at preschool	0–20	3.26	2.63	0.65
Externalizing problems at preschool	0–20	4.98	3.19	0.74
Internalizing problems at first grade	0–20	3.81	3.00	0.71
Externalizing problems at first grade	0–20	5.13	3.30	0.77

Abbreviations: Standard Deviation (SD); Cronbach’s Alpha (α).

**Table 2:** Participant characteristics at preschool (T1).

Participant Characteristics	Total (*n* = 1668)		Boys (*n* = 853)		Girls (*n* = 815)	
	*n*	%	*n*	%	*n*	%
Family structure						
Single-parent family	105	6.3	60	7.0	45	5.5
Two-parent family	1563	93.7	793	93.0	770	94.5
Attending institution						
Nursery school	883	52.9	451	52.9	432	53.0
Kindergarten	785	47.1	402	47.1	383	47.0
Annual household income (in millions of JPY)						
<3	165	10.2	97	11.7	68	8.6
3–5	711	43.8	365	44.0	346	43.5
6–8	467	28.7	225	27.1	242	30.4
≥9	282	17.4	143	17.2	139	17.5
Maternal education level						
Compulsory education	41	2.5	19	2.2	22	2.7
Upper secondary school	400	24.2	208	24.6	192	23.8
Up to four years at college/university	671	40.6	355	42.0	316	39.2
More than four years at college/university	540	32.7	264	31.2	276	34.2
Paternal education level						
Compulsory education	80	5.0	40	5.0	40	5.1
Upper secondary school	386	24.2	193	24.0	193	24.5
Up to four years at college/university	236	14.8	131	16.3	105	13.3
More than four years at college/university	891	55.9	441	54.8	450	57.1

Abbreviations: Japanese Yen (JPY).

**Table 3:** Participant characteristics at preschool (T1) and behavioral problems in first grade (T2).

Participant Characteristics	Total						Boys						Girls					
	Internalizing Problems			Externalizing Problems			Internalizing Problems			Externalizing Problems			Internalizing Problems			Externalizing Problems		
	Mean	SD	*p*	Mean	SD	*p*	Mean	SD	*p*	Mean	SD	*p*	Mean	SD	*p*	Mean	SD	*p*
Sex																		
Boys	3.78	2.96	0.612	5.7	3.42	<0.001 ***	-	-	-	-	-	-	-	-	-	-	-	-
Girls	3.85	3.04		4.50	3.05		-	-		-	-		-	-		-	-	
Family structure																		
Single-parent family	4.31	3.33	0.044 *	6.18	4.02	0.006 **	3.95	3.23	0.646	6.41	3.98	0.176	4.78	3.42	0.036 *	5.89	4.09	0.022 *
Two-parent family	3.78	2.98		5.06	3.24		3.77	2.94		5.68	3.37		3.80	3.01		4.42	2.96	
Attending institution																		
Nursery school	3.94	2.99	0.048 *	5.35	3.38	0.004 **	3.91	2.95	0.169	5.96	3.47	0.039 *	3.97	3.04	0.228	4.71	3.16	0.036 *
Kindergarten	3.67	3.00		4.88	3.20		3.63	2.97		5.47	3.35		3.72	3.04		4.26	2.90	
Annual household income (in millions of JPY)																		
<3	4.59	3.41	<0.001 ***	5.85	3.58	<0.001 ***	4.79	3.29	0.006 **	6.18	3.51	0.135	4.30	3.58	0.002 **	5.40	3.65	<0.001 ***
3–5	3.96	2.99		5.31	3.25		3.72	2.88		5.82	3.30		4.22	3.09		4.78	3.10	
6–8	3.55	2.87		4.99	3.16		3.62	2.85		5.67	3.38		3.50	2.90		4.36	2.79	
≥9	3.46	2.95		4.37	3.21		3.60	3.13		5.19	3.54		3.30	2.75		3.52	2.59	
Maternal education level																		
Compulsory education	5.44	3.80	<0.001 ***	7.00	3.98	<0.001 ***	5.56	3.84	0.002 **	7.67	4.24	<0.001 ***	5.34	3.86	<0.001 ***	6.45	3.76	0.001 **
Upper secondary	4.20	3.01		5.65	3.37		3.84	2.76		6.39	3.34		4.58	3.21		4.85	3.23	
Up to four years at college/university	3.87	2.97		5.10	3.27		4.02	3.04		5.68	3.33		3.70	2.89		4.46	3.07	
More than four years at college/university	3.32	2.84		4.62	3.11		3.32	2.90		5.12	3.38		3.32	2.80		4.13	2.75	
Paternal education level																		
Compulsory education	4.58	3.59	0.040 *	5.91	3.54	<0.001 ***	4.53	3.04	0.448	6.53	3.52	<0.001 ***	4.63	4.10	0.215	5.30	3.50	0.079
Upper secondary school	3.93	3.07		5.56	3.36		3.74	3.09		6.49	3.42		3.74	2.99		4.63	3.03	
Up to four years at college/university	3.74	3.04		5.35	3.29		3.78	3.01		5.94	3.37		4.11	3.15		4.62	3.04	
More than four years at college/university	3.72	2.89		4.71	3.13		3.73	2.90		5.21	3.29		3.71	2.89		4.23	2.89	

Abbreviations: Standard Deviation (SD); *p*-value (*p*); * significance at *p* < 0.05; ** significance at *p* < 0.01; *** significance at *p* < 0.001.

**Table 4:** Effect of parenting practices at preschool (T1) on behavioral problems at first grade (T2) in both boys and girls.

Parenting Practices	Model 1 Laxness				Model 2 Over-Reactivity				Model 3 Laxness and Over-Reactivity			
	B	SE	β	*p*	B	SE	β	*p*	B	SE	β	*p*
Internalizing Problems												
Laxness	0.144	0.081	0.037	0.075	-	-	-	-	0.133	0.082	0.034	0.107
Over-reactivity	−	−	−	−	0.111	0.057	0.040	0.052	0.095	0.058	0.035	0.102
Adjusted R^2^	0.363				0.370				0.369			
Externalizing Problems												
Laxness	0.193	0.078	0.046	0.013 *	-	-	-	-	0.168	0.079	0.040	0.034 *
Over-reactivity	−	−	−	−	0.178	0.057	0.060	0.002 **	0.164	0.058	0.055	0.005 **
Adjusted R^2^	0.493				0.496				0.498			

Note: Internalizing problems: Controlled for sex, family structure, attending institution, annual household income, maternal education level, paternal education level, and T1 internalizing problems. Externalizing problems: Controlled for sex, family structure, attending institution, annual household income, maternal education level, paternal education level, and T1 externalizing problems. Abbreviations: Unstandardized coefficient (B); Standard Error (SE); Standardized coefficient (β); *p*-value (*p*); * significance at *p* < 0.05; ** significance at *p* < 0.01.

**Table 5:** Effect of parenting practices at preschool (T1) on behavioral problems at first grade (T2) boys only.

Parenting Practices	Model 1 Laxness				Model 2 Over-Reactivity				Model 3 Laxness and Over-Reactivity			
	B	SE	β	*p*	B	SE	β	*p*	B	SE	β	*p*
Internalizing Problems												
Laxness	0.177	0.113	0.046	0.111	-	-	-	-	0.182	0.115	0.047	0.112
Over-reactivity	−	−	−	−	0.106	0.081	0.038	0.192	0.088	0.082	0.032	0.285
Adjusted R^2^	0.369				0.375				0.380			
Externalizing Problems												
Laxness	0.244	0.113	0.055	0.028 *	-	-	-	-	0.218	0.115	0.049	0.038 **
Over-reactivity	-	-	-	-	0.170	0.085	0.054	0.042 *	0.153	0.086	0.048	0.047 **
Adjusted R^2^	0.504				0.505				0.507			

Note: Internalizing problems: Controlled for family structure, attending institution, annual household income, maternal education level, paternal education level, and T1 internalizing problems. Externalizing problems: Controlled for family structure, attending institution, annual household income, maternal education level, paternal education level, and T1 externalizing problems. Abbreviations: Unstandardized coefficient (B); Standard Error (SE); Standardized coefficient (β); *p*-value (*p*); * significance at *p* < 0.05; ** significance at *p* < 0.01.

**Table 6:** Effect of parenting practices at preschool (T1) on behavioral problems at first grade (T2) girls only.

Parenting Practices	Model 1 Laxness				Model 2 Over-Reactivity				Model 3 Laxness and Over-Reactivity			
	B	SE	β	*p*	B	SE	β	*p*	B	SE	β	*p*
Internalizing Problems												
Laxness	0.119	0.117	0.030	0.308	-	-	-	-	0.088	0.119	0.022	0.460
Over-reactivity	-	-	-	-	0.119	0.080	0.044	0.138	0.103	0.082	0.038	0.210
Adjusted R^2^	0.366				0.376				0.385			
Externalizing Problems												
Laxness	0.142	0.108	0.037	0.183	-	-	-	-	0.117	0.109	0.031	0.282
Over-reactivity	−	−	−	−	0.188	0.076	0.071	0.014 *	0.178	0.078	0.067	0.023 *
Adjusted R^2^	0.456				0.464				0.474			

Note: Internalizing problems: Controlled for family structure, attending institution, annual household income, maternal education level, paternal education level, and T1 internalizing problems. Externalizing problems: Controlled for family structure, attending institution, annual household income, maternal education level, paternal education level, and T1 externalizing problems. Abbreviations: Unstandardized coefficient (B); Standard Error (SE); Standardized coefficient (β); *p*-value (*p*); * significance at *p* < 0.05.
